# The Evolving Role of Chemotherapy in the Management of Pleural Malignancies: Current Evidence and Future Directions

**DOI:** 10.3390/cancers17132143

**Published:** 2025-06-25

**Authors:** Yuliya Semenova, Zhandos Burkitbayev, Nurtas Kalibekov, Alexandr Digay, Bakhyt Zhaxybayev, Oxana Shatkovskaya, Saule Khamzina, Dinara Zharlyganova, Zhuldyz Kuanysh, Almira Manatova

**Affiliations:** 1 Department of Surgery, School of Medicine, Nazarbayev University, Astana 010000, Kazakhstan; yuliya.semenova@nu.edu.kz; 2 Chairman of the board, National Research Oncology Center, Astana 020000, Kazakhstan; zhan11@mail.ru; 3 Department of Multidisciplinary Surgery, National Research Oncology Center, Astana 020000, Kazakhstan; nurtas_1985@mail.ru (N.K.); aleksandr_digai@mail.ru (A.D.); mir-bahit@mail.ru (B.Z.); khamzina.saule88@gmail.com (S.K.); 4 Vice-Chairman of the board for Strategic Development, Scientific and Educational Activities, National Research Oncology Center, Astana 020000, Kazakhstan; 1972arty@mail.ru; 5 Department of Scientific Management, National Research Oncology Center, Astana 020000, Kazakhstan; science.nroc@gmail.com (D.Z.); zhuldyzkuanysh@icloud.com (Z.K.)

**Keywords:** malignant pleural mesothelioma, pleural metastases, malignant pleural effusion, chemotherapy, targeted therapy, immunotherapy, hyperthermic intrathoracic chemotherapy, pressurized intrathoracic aerosol chemotherapy

## Abstract

Pleural malignancies represent a clinically devastating group of oncological disorders, most commonly arising from metastatic disease, with lung and breast cancers being the most frequent primary sites. Malignant pleural mesothelioma is a primary malignancy of the pleura and occurs less often than metastatic pleural disease. Pleural malignancies often present with malignant pleural effusion, which typically indicates advanced-stage disease and is associated with poor overall prognosis. Treatment of pleural malignancies includes both palliative and definitive approaches. Palliative interventions primarily aim to relieve symptoms and improve quality of life. Definitive treatments include systemic chemotherapy, targeted therapy, and immunotherapy, depending on the type and molecular profile of the underlying tumor. In mesothelioma, platinum-based chemotherapy in combination with pemetrexed remains the cornerstone of treatment, while the combination of nivolumab and ipilimumab is recommended as first-line therapy for unresectable disease. For metastatic disease, systemic therapy is typically tailored to the primary tumor’s characteristics. Intrapleural administration of chemotherapeutic agents is one of the therapeutic strategies and hyperthermic intrathoracic chemotherapy and pressurized intrathoracic aerosol chemotherapy are the most recent innovations that are under active investigation. This review provides an up-to-date synthesis of systemic chemotherapy strategies for pleural malignancies, their integration with targeted and immune-based therapies, and recent advances in intrapleural chemotherapy modalities. It also explores existing knowledge gaps and outlines directions for future research and potential changes in clinical practice.

## Simple Summary

Pleural malignancies, often resulting from metastatic cancers like lung or breast cancer, are aggressive and typically present with malignant pleural effusion, indicating advanced disease and poor prognosis. Malignant pleural mesothelioma, a rarer primary pleural cancer, is also associated with a severe clinical course. Treatment strategies include palliative care to relieve symptoms and improve quality of life, as well as definitive therapies such as systemic chemotherapy, targeted therapy, and immunotherapy. Standard treatments for mesothelioma involve platinum-based chemotherapy with pemetrexed and immunotherapy for unresectable cases. Systemic therapy for metastatic disease is based on the tumor’s origin and profile. Intrapleural chemotherapy, including hyperthermic intrathoracic chemotherapy and pressurized intrathoracic aerosol chemotherapy, represents a promising area under investigation. This review summarizes current therapeutic approaches, recent advances, and highlights gaps in knowledge to guide future research and clinical innovation.

## 1. Introduction

Malignant pleural disease (MPD) represents a relatively rare but clinically devastating group of malignancies. MPD most commonly arises from pleural metastases of other cancers, with lung and breast cancers being the most frequent primary sites [1]. Malignant pleural mesothelioma (MPM), although the most common primary malignancy of the pleura, is considerably less prevalent than metastatic pleural disease [2]. Pleural malignancies frequently present with malignant pleural effusion (MPE), characterized by the presence of malignant cells in the pleural fluid. MPE is commonly associated with the accumulation of fluid in the pleural cavity and often signifies advanced malignancy [3].

While the global incidence of MPD is difficult to quantify precisely, it is estimated that approximately one million people develop the condition each year worldwide [4]. In the United States (US), approximately 150,000 people are diagnosed with MPE annually [5], while in the United Kingdom, this figure is around 50,000 [6]. The presence of MPE has been shown to nearly triple healthcare costs compared to those associated with the management of other malignancies [7]. Given that up to 15% of cancers present with MPE at the time of diagnosis [8], its clinical and economic impact is substantial.

The incidence of MPE has increased over the past decade. This upward trend is attributed to population aging and increased cancer survival rates, leading to a higher prevalence of advanced-stage malignancies that can result in MPE [9]. In resource-poor settings, delayed diagnosis of cancers commonly associated with MPE remains a significant challenge. This delay contributes to a higher burden of MPE due to more patients presenting with advanced-stage disease [10]. Although the incidence of MPM has stabilized or declined in many developed countries following asbestos bans, asbestos remains in use in numerous developing countries. This ongoing exposure raises concern about a future increase in MPM incidence in these settings [11].

Both palliative and definitive treatment approaches exist for MPE. Palliative interventions aim to relieve symptoms and improve quality of life, especially in patients with limited life expectancy [12]. In contrast, definitive treatments targeting the underlying malignancy can reduce pleural effusion formation in select patients, particularly those with chemosensitive tumors [13]. The scope of definitive treatment includes systemic chemotherapy, targeted therapy, and immunotherapy, depending on the type and molecular profile of the primary malignancy [14]. Intrapleural administration of chemotherapeutic agents has recently emerged as a promising strategy, with several modalities such as hyperthermic intrathoracic chemotherapy (HITOC) and pressurized intrathoracic aerosol chemotherapy (PITAC) showing potential benefits [15]. This comprehensive review aims to provide an up-to-date overview of systemic chemotherapy for MPD, including its combination with targeted therapy and immunotherapy. In addition, it highlights recent advances in intrapleural chemotherapy. This review also explores existing knowledge gaps and suggests directions for future research and potential changes in clinical practice.

## 2. Methodology of Study Search and Selection

A comprehensive literature search was conducted to fulfill the objectives of this review. Major databases relevant to evidence-based medicine were utilized, including Web of Science, ScienceDirect, and MEDLINE/PubMed. These databases were selected due to their extensive coverage of peer-reviewed studies in the fields of oncology and cancer care. The search strategy aimed to identify relevant literature addressing the scope of this review. The following terms were used either as keywords or controlled vocabulary (MeSH terms in PubMed), and combined using Boolean operators: “malignant pleural disease” OR “malignant pleural involvement” OR “pleural neoplastic disease” OR “pleural malignancy” OR “pleural cancer” OR “malignant pleural effusion” OR “malignant pleural mesothelioma” OR “pleural metastases” OR “pleural metastatic disease” AND “chemotherapy” OR “combination chemotherapy” AND “molecular targeted therapies” AND “immunotherapy” AND “cancer chemotherapy, regional perfusion” OR “intrapleural infusion” OR “intrapleural chemotherapy” OR “hyperthermic intrathoracic chemotherapy” OR “pressurized intrathoracic aerosol chemotherapy.” For MEDLINE/PubMed searches, the MeSH database was consulted to verify the appropriateness of the keywords and ensure that relevant synonyms, variations, and combinations were included.

All retrieved studies were subjected to a critical appraisal to assess their relevance and methodological quality. An inductive thematic analysis approach was employed to evaluate and synthesize the primary data from the identified studies. The extracted evidence was then organized into key thematic categories to enhance understanding of chemotherapy strategies for MPD. These categories included (1) available systemic chemotherapy regimens; (2) combination therapies involving targeted agents and immunotherapies; and (3) localized intrapleural chemotherapy techniques. The key findings of the included studies were critically analyzed and synthesized to provide an up-to-date overview of current clinical practice and future directions. Particular emphasis was placed on evaluating clinical effectiveness, safety profiles, and the integration of these therapies into multimodal treatment strategies aimed at improving patient outcomes.

## 3. Pathophysiology and Classification of Malignant Pleural Disease

In metastatic pleural disease, malignant involvement of the pleura may occur through direct invasion from adjacent cancerous structures or via lymphatic or hematogenous dissemination. In contrast, MPM typically presents with diffuse infiltration of the pleura by tumor cells [16]. MPD often manifests as MPE, characterized either by significant pleural fluid accumulation—commonly referred to as “wet MPE”—or by minimal or absent fluid, referred to as “dry MPE.” This distinction is influenced by differences in the extent of pleural involvement, the biological behavior of the causative tumor, and the balance between pleural fluid production and lymphatic drainage [17]. A thorough understanding of MPE pathophysiology is essential for accurately differentiating between these two clinical presentations of MPD.

### 3.1. Pathogenesis of Malignant Pleural Effusion

Wet MPE largely develops due to disturbances in Starling forces, which govern fluid movement across the pleural membranes. Alterations in hydrostatic and oncotic pressures play a central role in this process. Elevated hydrostatic pressure in the parietal pleural capillaries—often resulting from tumor-induced venous obstruction or lymphangitic spread—drives fluid out of the capillaries and into the pleural space. Conversely, a reduction in plasma oncotic pressure, commonly seen in malignancy-associated hypoalbuminemia, diminishes the reabsorptive force that would normally pull fluid back into the vascular compartment. The imbalance between these forces favors net fluid accumulation in the pleural cavity [17].

Increased vascular permeability and lymphatic obstruction due to tumor-related inflammation also contribute to fluid accumulation in the pleural cavity [18]. Tumor cells within the pleural space can release pro-inflammatory cytokines and growth factors—such as vascular endothelial growth factor (VEGF), tumor necrosis factor-alpha (TNF-α), and osteopontin —as well as proteolytic enzymes including urokinase-type plasminogen activator [19] and cathepsins B and D [20]. These factors increase vascular permeability, promote neoangiogenesis, and remodel the pleural membrane. Furthermore, direct pleural invasion by malignant cells disrupts the mesothelial barrier and impairs normal fluid reabsorption [17]. In some cases, malignant effusions may also contain cellular and molecular elements that actively inhibit fluid clearance mechanisms, compounding exudate accumulation [16].

However, fluid accumulation is not always observed in MPE; some tumors lead to substantial effusion, while others do not. The proportion of pleural malignancies presenting with effusion has been reported to range from 42% to 77% [17]. In general, tumors with a more localized or non-inflammatory character may not induce the same degree of fluid transudation. Examples of such tumors include non-small cell lung cancer (NSCLC), well-differentiated MPM, and lung carcinoid tumors, which are often slow-growing and less aggressive [21]. Pleural fibrosis or thickening may also impair fluid production or enhance its reabsorption. In certain cancers, such as MPM, mesothelial cells—responsible for producing pleural fluid—can be destroyed or replaced by fibrotic tissue, reducing fluid production [22]. Furthermore, tumors with dense connective tissue or fibrous stroma—such as benign fibrous tumors—may not lead to significant fluid accumulation due to the mechanical resistance provided by the surrounding tissues. This resistance can limit vascular leakage and maintain normal fluid dynamics [23]. Finally, the absence of pleural fluid at the time of MPE diagnosis may indicate early-stage disease. Dry MPE lacks the obvious physical signs of pleural effusion—such as dyspnea or chest discomfort caused by fluid buildup—potentially allowing for more effective curative interventions.

### 3.2. Classification of Tumors Causing Malignant Pleural Disease

The classification of pleural tumors can be organized into several schemes, depending on factors such as tumor origin, histopathological characteristics, and the extent of disease involvement. Based on tumor origin, all pleural tumors fall into two broad categories: primary pleural tumors, which arise directly from the pleural tissue, and metastatic pleural tumors, which result from the spread of malignancy from distant primary sites. In clinical practice, metastatic pleural tumors are significantly more common than primary ones. Among all primary tumor sites, lung cancer is the most frequent cause of metastatic pleural involvement, followed by breast cancer [1]. Other malignancies that may lead to MPE include ovarian cancer, thymic cancer, gastrointestinal cancers, lymphomas, and leukemias [16,17].

#### 3.2.1. Histological Classification of Pleural Tumors

According to the *2021* WHO Classification of Thoracic Tumors, all primary pleural tumors are categorized into mesothelial and hematolymphoid neoplasms. Mesothelial tumors are further subdivided into benign and preinvasive lesions, which include the adenomatoid tumor, well-differentiated papillary mesothelial tumor, and mesothelioma in situ, as well as invasive mesotheliomas. Mesotheliomas are classified into localized and diffuse forms, with the localized type typically associated with a better prognosis when completely resected [24]. There are three main histologic subtypes of mesothelioma: epithelioid, sarcomatoid, and biphasic. The epithelioid and sarcomatoid variants exhibit distinct architectural patterns, cytological features, and stromal characteristics, while the biphasic variant represents a combination of both histologic components [25].

Hematolymphoid tumors of the pleura are represented primarily by lymphomas, with two main subtypes described: primary effusion lymphoma (PEL) and diffuse large B-cell lymphoma (DLBCL) associated with chronic inflammation. PEL is typically not associated with solid tumor formation, whereas DLBCL associated with chronic inflammation presents as a mass-forming lymphoma. Primary pleural lymphomas are exceedingly rare, unlike secondary pleural involvement seen in disseminated lymphomas. Other, even rarer types of primary pleural lymphoma include follicular lymphoma, extranodal marginal zone lymphoma, and anaplastic large cell lymphoma [25].

As for metastatic disease, it commonly displays cytomorphological features of the primary tumor from which it originates. However, immunohistochemistry is often required to differentiate it from MPM and to suggest the site of origin [25].

#### 3.2.2. Staging of Pleural Tumors

The staging of MPM follows the TNM (Tumor, Node, Metastasis) classification system developed by the American Joint Committee on Cancer (AJCC) and the Union for International Cancer Control, with the current guidelines detailed in the 8th edition of the *AJCC Cancer Staging Manual*. According to this system, T1 refers to a tumor limited to the ipsilateral parietal pleura, with or without involvement of the visceral pleura. T2 describes a tumor involving all ipsilateral pleural surfaces with invasion into the diaphragm or lung. T3 indicates a tumor involving all ipsilateral pleural surfaces with extension into the endothoracic fascia, mediastinal fat, or a solitary focus of chest wall or pericardial involvement without full-thickness invasion. T4 is characterized by unresectable disease with diffuse or multifocal chest wall invasion, involvement of rib(s), mediastinal organ(s), spine or brachial plexus, peritoneum, contralateral pleura, or extensive pericardial involvement. Lymph node involvement is categorized as N0 (no regional lymph node metastases), N1 (metastases in ipsilateral bronchopulmonary, hilar, or mediastinal lymph nodes), and N2 (metastases in subcarinal or contralateral bronchopulmonary, hilar, or mediastinal lymph nodes). Distant metastasis is classified as M0 (no distant metastases) or M1 (presence of distant metastases) [26]. From a clinical perspective, T1N0M0 corresponds to stage IA; T2–T3N0M0 to stage IB; T1–T2N1M0 to stage II; T3N1M0 to stage IIIA; any T4 or N2 disease with M0 to stage IIIB; and any M1 disease to stage IV [25].

Metastatic pleural disease is also staged using the TNM classification. Pleural spread from primary lung or thymic carcinoma is considered M1a disease. In contrast, pleural metastases originating from other primary tumors are staged as distant metastases (M1), based on the staging criteria of the respective primary tumor [25].

Lymphoid tumors of the pleura are staged using the Ann Arbor system with Cotswolds modifications [27]. If the pleura is the only involved site, the disease is classified as stage IE (extranodal disease limited to one site). In cases where pleural disease is accompanied by regional lymph node involvement, the disease is staged as stage IIE. PEL, however, is typically an aggressive neoplasm presenting with diffuse involvement of body cavities without associated nodal disease, and is therefore usually considered stage IV at diagnosis [25].

## 4. Management of Malignant Pleural Disease

The management of MPD needs to be tailored to individual patient factors and account for the disease stage, patient performance status, symptom burden, lung re-expansion potential, expected survival, and patient preferences.

### 4.1. Diagnosis

In patients with MPD, the majority of those with fluid accumulation (wet MPE) present with symptoms, making the diagnosis relatively straightforward when an underlying malignancy has already been established. However, because the clinical signs of MPE are non-specific and can occur in both malignant and non-malignant conditions, newly developed pleural effusion in patients with risk factors for cancer should be carefully evaluated. Imaging of the pleura is a fundamental step in the diagnostic workup of MPD. Nevertheless, a definitive diagnosis of MPD requires the identification of malignant cells in pleural fluid cytology or pleural tissue biopsy [28].

Chest radiography is a basic imaging technique for MPE capable of detecting pleural effusions as small as 50 mL on a lateral chest film and 100–200 mL on an anteroposterior chest film [29]. However, chest computed tomography (CT) remains the gold standard imaging modality for the diagnosis of MPE. CT scanning allows for detailed evaluation of effusion size, the presence of loculations, and pleural thickening [30]. Chest CT has a high specificity for MPE, ranging from 78% to 100%, depending on the study, although its sensitivity is lower, reported between 36% and 68% [28]. More recently, chest ultrasound has emerged as a valuable tool for detecting pleural nodularity, identifying loculations that may complicate pleural procedures, and mapping the location of intercostal vessels. It is now considered best practice to perform all invasive diagnostic pleural procedures under ultrasound guidance [30]. Positron emission tomography (PET) has an estimated specificity of 57% to 74% and a sensitivity of 70% to 93% for MPD. Due to the possibility of false-positive results, PET is primarily used for staging rather than for the initial detection of MPD [28].

Thoracentesis with subsequent cytological examination of aspirated pleural fluid is the most commonly used initial method for evaluating suspected MPD. However, its diagnostic sensitivity varies significantly depending on the underlying malignancy. In general, adenocarcinomas demonstrate the highest sensitivity, ranging from 70% to 79%, whereas MPM shows markedly lower sensitivity, typically between 5% and 10% [28].

Video-assisted thoracoscopic surgery (VATS) remains the gold standard for establishing a definitive diagnosis of MPD. It enables direct visualization of the pleural cavity and facilitates the collection of multiple high-quality biopsies under direct vision, yielding a diagnostic accuracy exceeding 90% in most studies [31].

Percutaneous pleural biopsy, commonly performed using a needle, is another diagnostic option with a reported sensitivity ranging from 40% to 74%. Its diagnostic yield improves significantly with ultrasound guidance, particularly when a focal pleural abnormality can be visualized and targeted. Although less invasive than VATS, percutaneous biopsy is typically reserved for patients who are not suitable candidates for surgical procedures or in settings where thoracoscopy is unavailable [28]. 

### 4.2. Multimodal Treatment Approaches

At the time of presentation, MPD is often associated with MPE, which typically indicates advanced-stage disease and a poor overall prognosis. The treatment of MPE aims to relieve symptoms, improve quality of life, and minimize the need for repeated interventions. In general, treatment strategies for MPE can be broadly categorized into systemic and procedural approaches [32]. This subsection will primarily focus on procedural interventions, while systemic treatments will be discussed in detail in subsequent sections of the review.

Dyspnea is the most common presenting symptom of MPE, and many treatment strategies focus on alleviating it. Therapeutic thoracentesis is typically the first-line intervention. It is recommended that the initial thoracentesis be performed with a large-volume drainage to assess whether fluid removal improves dyspnea and to evaluate the lung’s ability to re-expand. This assessment guides the selection of further management strategies [28]. Although repeated thoracentesis may be necessary due to recurrent symptoms, it is not considered the standard of care for MPE management for several reasons: (1) increased risk of procedure-related complications; (2) greater patient dependence on the healthcare system; and (3) promotion of pleural inflammation with subsequent loculation [33]. Therefore, the preferred approach is to perform a definitive palliative pleural procedure—such as pleurodesis or the placement of an indwelling pleural catheter (IPC)—following the initial large-volume thoracentesis [34].

Pleurodesis involves instilling a sclerosing agent into the pleural cavity to induce pleural inflammation and subsequent fibrosis, thereby preventing fluid re-accumulation. Several chemical agents have been proposed for this purpose; however, talc slurry and talc poudrage remain the most commonly used agents due to their effectiveness, low cost, and relatively favorable side-effect profile. The most recent meta-analysis demonstrated comparable pleurodesis failure rates between the two techniques, as well as similar incidences of adverse events, including pleural empyema and pneumonia [35]. 

Indwelling pleural catheters are tunneled catheters inserted into the pleural cavity that allow intermittent drainage of pleural fluid in the outpatient setting. Spontaneous pleurodesis occurs in approximately 24–58% of cases following IPC placement [28]. The Cochrane Collaboration conducted a network meta-analysis to evaluate the comparative effectiveness of various definitive palliative pleural procedures. Talc pleurodesis was found to have the highest effectiveness in terms of lower failure rates. Although IPCs are associated with lower rates of definitive pleurodesis compared to talc pleurodesis, they offer comparable control of dyspnea [36].

Cytodestructive surgery plays a limited role in the management of metastatic pleural disease and is primarily reserved for selected patients with MPM. The main surgical procedures include extrapleural pneumonectomy and lung parenchyma-preserving pleurectomy/decortication. These procedures are typically not indicated for patients with metastatic cancers, as the disease is often in an advanced stage, and extrapleural pneumonectomy, in particular, is associated with high rates of complications, morbidity, and mortality [32]. Extrapleural pneumonectomy has not demonstrated a survival benefit even in patients with MPM and has largely been abandoned following the publication of the Mesothelioma and Radical Surgery (MARS) trial, which highlighted its lack of efficacy and associated risks [37]. Being a minimally invasive technique, VATS may be employed for pleural decortication, particularly in cases of unexpandable lung or complex, loculated effusions [38].

Systemic therapy—including chemotherapy, targeted therapy, or immunotherapy—can contribute to the control of MPD, depending on the patient’s performance status, treatment tolerability, and overall suitability. In metastatic pleural disease, systemic therapy is primarily palliative, whereas in MPM, it may have a potentially curative role if the disease is diagnosed at an early stage [39]. Localized intrapleural chemotherapy has emerged as a promising modality, offering the advantage of achieving high drug concentrations at the tumor site while minimizing systemic toxicity [32]. Targeted therapies and immunotherapies represent additional therapeutic opportunities in select patient populations, although their roles in MPD are still under investigation [4]. Given the rapid evolution of systemic and localized treatments, further research is warranted to define their optimal application in the management of MPD.

## 5. Systemic Chemotherapy of Malignant Pleural Disease

### 5.1. Systemic Chemotherapy of Malignant Pleural Mesothelioma

A number of clinical trials were conducted during the 1990s to evaluate the effectiveness of single-agent chemotherapy regimens in the treatment of MPM. These regimens typically included platinum analogues, multitargeted antifolate chemotherapy agents, nucleoside analogues, and alkylating agents. The response rates were generally low, ranging from 0% to 13%, as were progression-free survival (PFS) (2–5 months) and median overall survival (OS) (5–8 months). Among all single-agent regimens tested, cisplatin, platinum analogue, demonstrated slightly better response rates of around 20% [40].

The addition of pemetrexed (a folate pathway inhibitor) to platinum-based therapy subsequently led to improved treatment outcomes. The EMPHACIS trial, the first phase III RCT to investigate this combination, was published by Vogelzang et al. in 2003 [41]. In this study, 456 patients were randomized to receive either cisplatin plus pemetrexed or cisplatin alone. The combination therapy was found to be superior, resulting in a longer median OS (12.1 months versus 9.3 months, *p* = 0.020), prolonged time to disease progression (5.7 months versus 3.9 months, *p* = 0.001), and higher objective response rates (41.3% versus 16.7%, *p* < 0.0001) [41]. The EORTC trial, another phase III RCT published in 2005, compared cisplatin plus raltitrexed (a thymidylate synthase inhibitor) versus cisplatin alone in a cohort of 250 patients. Similar to the findings from the EMPHACIS trial, the combination regimen resulted in an improved response rate (23.6% versus 13.6%, *p* = 0.056) as well as longer median OS (11.4 months versus 8.8 months) and one-year survival rates (46% versus 40%). However, the combination therapy was associated with approximately twice the rate of grade 3 or 4 toxicities, the most common being neutropenia and emesis [42].

Several trials have investigated the use of the platinum-based chemotherapy agent carboplatin in combination with pemetrexed. Castagneto et al. reported an overall response rate of 25%, comprising a 21% partial response rate and a 4% complete response rate [43]. Ceresoli et al. observed a disease control rate exceeding 60%, with a median time to progression of more than 7 months and a median OS of over 10 months [44]. Similarly, Katirtzoglou et al. reported a 29% partial response rate, stable disease in 54.9% of patients, and a median OS of 14 months [45]. The combination of pemetrexed and carboplatin was associated with a relatively high incidence of hematologic toxicities, with grade 3 events observed in 47.3% of patients and grade 4 events in 6.5% [43]. Although none of the aforementioned studies [43,44,45] directly compared carboplatin with other platinum-based agents, a non-randomized study by Santoro et al. compared pemetrexed plus cisplatin versus pemetrexed plus carboplatin in a large cohort of MPM patients [46]. This study found a higher incidence of grade 3–4 toxicities in the pemetrexed–carboplatin group compared to the pemetrexed–cisplatin group, with neutropenia being the most common adverse event (36.1% vs. 23.9%, respectively). Moreover, a slightly lower response rate was observed in the pemetrexed–carboplatin group (21.7%) compared to the pemetrexed–cisplatin group (26.3%) [46]. In clinical practice, cisplastin is often used as an alternative to carboplatin, particularly in patients unable to tolerate carboplatin-related toxicities [40].

Currently, a combination of pemetrexed and a platinum agent is considered the standard of care for patients with MPM [47]. A recent meta-analysis of RCTs reported a pooled PFS of 6.7 months and an OS of 14.2 months for the platinum–pemetrexed regimen. These outcomes were superior to those of other experimental chemotherapy regimens, which showed a pooled median OS of 13.5 months [48].

Gemcitabine (a nucleoside analogue) is another chemotherapy agent that was investigated in combination regimens for MPM. The first study evaluating this approach was conducted by Byrne et al. and published in 1999. In this study, a combination therapy involving gemcitabine achieved a 47.6% objective response rate (complete + partial responses), with a median response duration of 25 weeks and a median OS of 41 weeks [49]. A subsequent study by Lee et al. compared two combination therapies: gemcitabine with a platinum-based agent (cisplatin or carboplatin) versus pemetrexed with a platinum-based agent. This real-world analysis reported non-inferiority of the gemcitabine–platinum doublet compared to the pemetrexed–platinum doublet in terms of median OS, 1-year and 2-year survival rates, and toxicity profile [50]. Additionally, a combination of gemcitabine and pemetrexed was evaluated in clinical studies but was found to be clinically less effective than the standard combination of pemetrexed with a platinum-based agent [51].

Vinorelbine, a vinca alkaloid, has been investigated both as monotherapy and in combination regimens. The first study evaluating vinorelbine in MPM patients was conducted by Steele et al. and published in 2000. It reported a partial response rate of 24% and disease stabilization in 55% of patients [52]. Due to its favorable toxicity profile, vinorelbine has since been studied in combination with other chemotherapeutic agents, in both first-line and second-line treatment settings. Fennell et al. evaluated a combination of vinorelbine with the platinum analogue oxaliplatin as a first-line therapy. They reported a response rate of 23%, PFS of 4.7 months, 1-year survival rate of 27%, and OS of 8.8 months. Neutropenia was the most common grade 3–4 toxicity, occurring in 18% of patients [53]. In the relapsed disease setting, vinorelbine in combination with active symptom control was associated with an improvement in PFS (4.2 months) compared to active symptom control alone (2.8 months) [54].

Mitomycin C, doxorubicin, and cyclophosphamide were among the early chemotherapy agents evaluated for the treatment of MPM. However, clinical trials have demonstrated that these agents, whether used as monotherapy or in combination, provided limited clinical benefit. For instance, a phase II RCT conducted by the Cancer and Leukemia Group B investigating the combination of cisplatin with mitomycin C and doxorubicin reported only modest anti-tumor activity [55]. Similarly, a randomized trial comparing doxorubicin and cyclophosphamide found no significant differences in outcomes, with neither agent achieving tumor remission [56].

Patients with relapsed MPM represent a challenging population that often requires different chemotherapy strategies compared to chemotherapy-naïve patients. In certain cases, re-treatment with first-line agents—particularly pemetrexed—may be considered if the initial response was favorable and more than 12 months have elapsed since the original treatment [40]. However, second-line options more commonly include vinorelbine and gemcitabine, which have demonstrated modest disease control and manageable toxicity profiles. For instance, Fennell et al. reported a PFS of 4.2 months in patients treated with vinorelbine [54]. In another study, Zauderer et al. evaluated vinorelbine and gemcitabine as second- or third-line therapies and found that objective responses were rare, while the toxicity burden was considerable—46% of patients experienced at least one grade 3–4 adverse event [57].

Irinotecan, a topoisomerase I inhibitor, and lurbinectedin, a marine-derived alkaloid, have been explored as treatment options in the relapsed setting of MPM. Koda et al. evaluated the effectiveness and safety of irinotecan in combination with gemcitabine in a retrospective cohort study. Although the objective response rate was low (2.1%), the disease control rate reached 66.1%, and the regimen was generally well tolerated, with neutropenia being the most common grade 3–4 adverse event (12.9%) [58]. Lurbinectedin monotherapy was assessed in a phase II trial involving patients previously treated with platinum-pemetrexed chemotherapy with or without immunotherapy. The study reported a median PFS of 4.1 months and a median OS of 11.1 months. Grade 3–4 toxicities were observed in 50% of patients [59]. Table 1 summarizes the primary first-line, as well as second- and third-line, chemotherapy regimens for patients with mesothelioma.

### 5.2. Systemic Chemotherapy of Metastatic Pleural Disease

For metastatic pleural disease, the selection of a systemic chemotherapy regimen depends on the histological subtype and molecular profile of the primary tumor, as well as the patient’s performance status, treatment tolerability, and overall clinical suitability. Existing comorbidities also influence treatment decisions. In cases where the risk of systemic chemotherapy-associated adverse events significantly compromises the patient’s quality of life, supportive care becomes the preferred management strategy [4].

#### 5.2.1. Systemic Chemotherapy for Pleural Involvement in Lung Cancer

In metastatic pleural involvement secondary to lung cancer, treatment is further guided by whether the tumor is classified as NSCLC or small cell lung cancer (SCLC). Systemic chemotherapy remains a cornerstone of NSCLC management, particularly for patients without actionable driver mutations or with low expression of programmed death-ligand 1 (PD-L1) [60]. Platinum-based doublet chemotherapy is the standard first-line approach, typically involving a platinum compound (cisplatin or carboplatin) combined with a second cytotoxic agent. For nonsquamous NSCLC, pemetrexed is the preferred partner drug, whereas for squamous NSCLC, gemcitabine or a taxane (paclitaxel or docetaxel) is commonly used.

The first RCT evaluating the efficacy of the pemetrexed–cisplatin combination in advanced nonsquamous NSCLC was published in 2000. In this phase III trial, Manegold et al. reported a partial response rate of 39%, disease stabilization in 47% of patients, and a median OS of 10.9 months [61]. Subsequently, Scagliotti et al. compared the efficacy and safety of the pemetrexed–cisplatin regimen with that of gemcitabine–cisplatin. The median OS was equivalent in both treatment arms (10.3 months each), confirming the non-inferiority of the pemetrexed–cisplatin regimen. However, the pemetrexed–cisplatin combination demonstrated a more favorable safety profile, with grade 3–4 neutropenia occurring in only 15% of patients, compared to 27% in the gemcitabine–cisplatin group [62]. Pemetrexed was also evaluated in combination with carboplatin. In a study by Zukin et al., the pemetrexed–carboplatin doublet was compared to pemetrexed monotherapy. The combination regimen yielded superior outcomes in terms of overall response rate (23.8% vs. 10.3%), median PFS (5.8 months vs. 2.8 months), and median OS (9.3 months vs. 5.3 months) [63].

Patients with pleural metastases secondary to squamous NSCLC and good performance status may be treated with platinum-based doublet chemotherapy regimens. These typically consist of a platinum compound (cisplatin or carboplatin) combined with a second agent such as paclitaxel, gemcitabine, or vinorelbine [62,64,65]. Such regimens have been shown to improve OS and quality of life compared to best supportive care, as demonstrated by a meta-analysis of 16 RCTs [66]. In contrast, pemetrexed is not recommended for squamous NSCLC due to its lack of efficacy in this subtype, potentially attributed to higher thymidylate synthase expression in squamous tumors, which diminishes pemetrexed’s antitumor activity [67].

As for SCLC, the standard first-line regimen for patients with pleural metastases consists of a platinum agent (cisplatin or carboplatin) in combination with etoposide, a topoisomerase II inhibitor. This regimen has demonstrated efficacy in improving survival outcomes and is endorsed by multiple clinical practice guidelines and trials [68,69,70,71]. A meta-analysis of RCTs by Rossi et al. found no significant difference between cisplatin-based and carboplatin-based regimens in terms of median OS (9.6 months vs. 9.4 months, respectively; *p* = 0.37) or median PFS (5.5 vs. 5.3 months; *p* = 0.25). However, differences in toxicity profiles were noted: hematologic toxicities were more frequent with carboplatin, while non-hematologic toxicities were more common with cisplatin [72]. A pooled analysis of clinical trial data similarly found no significant difference in efficacy between cisplatin–etoposide and carboplatin–etoposide regimens but concluded that overweight and obese patients may derive more benefit from cisplatin–etoposide [73].

Other chemotherapy regimens for extensive-stage SCLC include combinations of a platinum agent with irinotecan or paclitaxel, or a triplet regimen of etoposide with cyclophosphamide and doxorubicin. A Japanese phase III trial initially reported superiority of the cisplatin–irinotecan regimen over cisplatin–etoposide in terms of survival [74], but subsequent international phase III trials failed to confirm this benefit [75,76]. A comparison between the cyclophosphamide–doxorubicin–etoposide regimen and the carboplatin–paclitaxel regimen demonstrated comparable outcomes in terms of overall response rate (60% vs. 61%) and PFS (4.9 vs. 5.2 months, respectively), although the former was associated with higher rates of hematologic toxicities [77]. Additionally, a trial comparing cisplatin–amrubicin (a synthetic anthracycline and topoisomerase II inhibitor) with cisplatin–irinotecan showed a modest inferiority of the amrubicin regimen in terms of median PFS (5.1 vs. 5.6 months) and response rate (72.3% vs. 77.9%), with significantly greater hematologic toxicity [78]. Overall, the platinum–etoposide combination remains the standard first-line chemotherapy regimen for patients with extensive stage-SCLC, including those with pleural involvement. Table 2 summarizes the commonly used systemic chemotherapy regimens for the treatment of pleural metastases in lung cancer.

#### 5.2.2. Systemic Chemotherapy for Pleural Involvement in Non-Lung Primary Cancers

Breast cancer is the second most common primary tumor associated with MPD after lung cancer. Anthracycline- and taxane-based regimens are standard first-line chemotherapy options for this patient group. Katsumata et al. conducted a phase III trial comparing three arms: doxorubicin plus cyclophosphamide (an anthracycline-based regimen), docetaxel monotherapy (a taxane), and an alternating schedule of both regimens. The study found no significant differences in median time to treatment failure among the groups, but OS was superior in the docetaxel monotherapy arm [79]. This finding was supported by a subsequent meta-analysis of RCTs, which reported that neither taxane–anthracycline combination regimens nor anthracycline-based regimens significantly improved time to progression, objective response rate, or disease control rate compared to taxane monotherapy [80]. A comprehensive review of RCTs also found that while taxane–anthracycline combinations yielded higher overall and complete response rates than anthracycline-based regimens alone, they did not confer a survival advantage and were associated with significantly higher rates of grade 3–4 toxicities [81].

Anthracyclines and taxanes are recommended for use in MPD secondary to breast cancer if they have not been previously administered, or if at least one year has elapsed since their last use. Alternative chemotherapy regimens include vinorelbine, gemcitabine, and platinum-based combinations [82]. Gennatas et al. evaluated the efficacy and safety of a gemcitabine–vinorelbine regimen in patients previously treated with anthracyclines and/or taxanes. The study reported an overall response rate of 36%, with a median duration of response of 7 months and median OS of 14 months. The combination was well tolerated, supporting its utility in this treatment setting [83]. Additionally, a retrospective cohort study reported that patients receiving platinum-based chemotherapy had a median OS of 6.13 months, and 40.5% of those with visceral crisis achieved clinical resolution. These findings suggest that platinum agents may offer a viable option for this patient category [84].

The pleural cavity constitutes the most common site of extra-abdominal metastases in ovarian cancer [28]. Systemic chemotherapy remains the cornerstone of treatment in such cases, particularly with taxane–platinum regimens. This approach typically involves a combination of paclitaxel and carboplatin and is supported by multiple RCTs demonstrating both efficacy and an acceptable safety profile. Consequently, clinical practice guidelines recommend paclitaxel–carboplatin as the primary adjuvant chemotherapy for all stages of ovarian cancer, as well as for recurrent platinum-sensitive disease and as neoadjuvant therapy in patients with bulky stage III/IV tumors—including those with pleural metastases—since it addresses both abdominal and extra-abdominal disease spread [85]. In cases of platinum-sensitive relapse or intolerance to paclitaxel, rechallenge with alternative platinum-based regimens such as carboplatin plus docetaxel [86] or carboplatin plus pegylated liposomal doxorubicin [87] may be considered.

MPD secondary to gastrointestinal cancers is relatively uncommon, accounting for approximately 5% of all MPD cases [88]. As with MPD caused by lung, breast, or ovarian cancers, its presence in gastrointestinal malignancies is associated with poor prognosis. In gastric cancer, systemic chemotherapy regimens such as FOLFOX (5-fluorouracil, leucovorin, and oxaliplatin) are frequently used [89]. However, there is a notable lack of clinical trials specifically evaluating the efficacy of systemic chemotherapy for MPD due to gastric cancer. In metastatic colorectal cancer, regimens such as FOLFOX or XELOX (capecitabine and oxaliplatin) are routinely used [90]. Similar to gastric cancer, there is a paucity of studies specifically investigating the effectiveness of systemic chemotherapy for managing pleural metastases in colorectal cancer.

Systemic chemotherapy for MPD secondary to thymic epithelial tumors has been explored in several studies. A study by the Japan Clinical Oncology Group evaluated the efficacy of dose-dense weekly chemotherapy in patients with advanced thymoma (stages IVa and IVb) and reported an overall response rate of 59% and a median PFS of 0.79 years [91]. Additionally, a retrospective analysis assessed the outcomes of systemic chemotherapy in patients with advanced thymic carcinoma. This study found that platinum-based doublet, triplet, and quadruplet chemotherapy regimens were associated with improved survival outcomes. The median survival time was 24.5 months, with an objective response rate of 47.7% and a disease control rate of 80.2%, indicating the potential efficacy of platinum-based regimens in managing this group of patients [92].

All lymphatic malignancies have the potential to develop MPD, with Hodgkin and non-Hodgkin lymphomas showing pleural involvement in approximately 20–30% of cases during the disease course [88]. Systemic chemotherapy remains the primary treatment modality for pleural metastases in lymphatic cancers, particularly lymphomas, and has been evaluated in multiple studies. Aoki et al. conducted a large multicenter retrospective study of patients with primary mediastinal large B-cell lymphoma (PMBCL) presenting with MPE. The study assessed outcomes associated with various systemic chemotherapy regimens, including CHOP (cyclophosphamide, doxorubicin, vincristine, and prednisolone), as well as second- and third-generation regimens (though specific components of these were not detailed). The reported 4-year OS was 67% for patients treated with CHOP and 91% for those receiving second- or third-generation regimens [93]. Table 3 provides an overview of systemic chemotherapy regimens used in MPD secondary to non-pulmonary primary cancers.

## 6. Novel Systemic Therapy Regimens for Malignant Pleural Disease

The development of novel therapies has significantly expanded the treatment options available for patients with MPD. Targeted therapies and immunotherapies have emerged as important adjuncts or alternatives to systemic chemotherapy, particularly in cases where cancers exhibit identifiable molecular drivers or immunogenic profiles. Although systemic chemotherapy remains the cornerstone of MPD treatment, these newer modalities provide additional therapeutic options in selected clinical contexts.

### 6.1. Targeted Therapy and Immunotherapy of Malignant Pleural Mesothelioma

A variety of targeted therapies have been investigated in patients with MPM, including inhibitors of VEGF, platelet-derived growth factor receptor alpha (PDGFR-α), epidermal growth factor receptor (EGFR), the phosphatidylinositol 3-kinase (PI3K)/AKT/mammalian target of rapamycin (mTOR) pathway (PI3K/AKT/mTOR), the fibroblast growth factor (FGF), and focal adhesion kinase (FAK), among others. These agents have yielded mixed results in clinical trials. Among immunotherapies for mesothelioma, immune checkpoint inhibitors (ICIs) targeting PD-1, PD-L1, and cytotoxic T-lymphocyte-associated protein (CTLA-4) have demonstrated the most promise. However, most of these therapeutic agents have not been incorporated into routine clinical practice, often due to limited clinical efficacy and safety concerns.

#### 6.1.1. Targeted Therapy Agents for Malignant Pleural Mesothelioma

Targeting angiogenesis in mesothelioma is a key therapeutic approach, given the tumor’s reliance on neovascularization. Numerous anti-VEGF agents have been investigated in combination with chemotherapy, but only bevacizumab has demonstrated efficacy in a phase III RCT. In the MAPS trial, Zalcman et al. reported a significant improvement in median OS in patients receiving bevacizumab in addition to the pemetrexed–cisplatin regimen, compared with chemotherapy alone (18.8 vs. 16.1 months; *p* = 0.0167). Although the combination was associated with a higher incidence of grade 3–4 toxicities (71% vs. 62%), these events were generally manageable [94].

Other anti-VEGF agents have also been evaluated in combination with chemotherapy in patients with mesothelioma. For instance, cediranib was studied in combination with pemetrexed and cisplatin in a phase II trial, showing promising results in terms of PFS [95]. Nintedanib also showed improved PFS in a phase II trial; however, the subsequent phase III study did not confirm this benefit [96]. A phase II trial combined axitinib with cisplatin and pemetrexed in chemotherapy-naïve MPM patients. While axitinib inhibited angiogenesis, there was no significant difference in PFS or OS compared to chemotherapy alone [97]. Ramucirumab combined with gemcitabine was studied as a second-line treatment in a phase II RCT conducted by Pinto et al., which showed a median OS of 13.8 months for the combination group compared to 7.5 months for gemcitabine alone [98]. These encouraging results are reflected in the American Society of Clinical Oncology (ASCO) guidelines, which recommend ramucirumab in combination with gemcitabine for patients with disease progression after treatment with pemetrexed and a platinum-based agent [99].

Targeting PDGFR is another therapeutic approach for patients with MPM, as this receptor is often overexpressed in mesothelioma tumors and contributes to tumor growth and angiogenesis. Moreover, many anti-PDGFR agents also have activity against VEGFR, further supporting their potential utility [40]. Vatalanib was studied in a phase II trial involving chemotherapy-naïve mesothelioma patients and demonstrated a median PFS of 4.1 months and a median OS of 10 months [100]. However, since the trial did not meet its primary endpoint, further development of vatalanib was not pursued. Another phase II RCT evaluated sorafenib in MPM patients, but the outcomes did not indicate substantial improvement in survival or disease control, thereby limiting its therapeutic promise in this setting [101]. Sunitinib was also assessed in a phase II trial in patients who had progressed following first-line chemotherapy and showed only modest activity; the findings were insufficient to support continued investigation [102]. Imatinib has been explored in combination with cisplatin and pemetrexed, but the regimen was poorly tolerated and showed limited efficacy [103]. Overall, clinical evidence supporting the efficacy of PDGFR inhibitors in MPM remains limited, with most data derived from early-phase clinical trials and preclinical studies.

EGFR inhibitors have been investigated in MPM due to the frequent overexpression of EGFR in mesothelioma tumors, reported in approximately 60–70% of cases [104]. However, similar to PDGFR inhibitors, the clinical outcomes of EGFR-targeted therapies have been largely disappointing. A phase II RCT evaluating gefitinib monotherapy in patients with mesothelioma failed to meet its primary endpoints of PFS and OS [105]. Erlotinib, another EGFR tyrosine kinase inhibitor, demonstrated similarly limited efficacy, with a lower OS rate observed in the erlotinib arm (40%) compared to the cisplatin–pemetrexed arm (50%) [106]. While cetuximab, an EGFR-targeting monoclonal antibody, has shown some potential in preclinical models [107], a subsequent phase II trial did not meet its primary endpoint of PFS at 18 months [108].

The PI3K/AKT/mTOR signaling pathway is frequently dysregulated in mesothelioma, contributing to tumor proliferation, survival, and therapeutic resistance [40]. Several clinical trials have investigated inhibitors targeting this pathway. Everolimus, an mTOR inhibitor, was evaluated in a phase II trial but failed to meet its primary endpoint: a PFS rate of ≥50%, with the observed PFS rate being only 29% [109]. LY3023414, an oral dual PI3K/mTOR inhibitor, was assessed in a phase I cohort expansion study. Administered at a dose of 200 mg twice daily, LY3023414 exhibited an acceptable safety profile. However, its clinical efficacy was limited, with an objective response rate of 2.4% and a median PFS of 2.8 months [110].

Mesotheliomas often exhibit overexpression of various FGFs and FGF receptors (FGFRs), leading to autocrine or paracrine activation of downstream signaling pathways that promote tumor growth and angiogenesis [40]. To improve therapeutic efficacy, FGFR inhibitors have been investigated in combination with standard chemotherapies such as pemetrexed and cisplatin. For instance, GSK3052230, a soluble decoy receptor that selectively binds FGFs, was evaluated in combination with pemetrexed and cisplatin in patients with unresectable MPM, yielding an overall response rate of 39% and a disease control rate of 86%. The combination was generally well tolerated, partly due to the structural features of GSK3052230 that reduce its affinity for certain FGFs, thereby limiting off-target effects [111]. Despite these initially encouraging results, GSK3052230 was not further evaluated in RCTs.

FAK, also known as protein tyrosine kinase 2, is a non-receptor cytoplasmic tyrosine kinase that plays a pivotal role in several cellular processes, including survival, proliferation, migration, and invasion [40]. The Control of Mesothelioma with MAiNtenance Defactinib (COMMAND) trial evaluated defactinib, a selective FAK inhibitor, as maintenance therapy following at least four cycles of first-line chemotherapy. In this placebo-controlled RCT, defactinib did not improve PFS or OS compared to placebo. The lack of efficacy was partially attributed to alterations in the tumor immune microenvironment induced by prior chemotherapy [112].

While most targeted therapies have shown limited benefit in MPM, there is growing interest in the potential of immunotherapy agents to improve patient outcomes.

#### 6.1.2. Immunotherapy Agents for Malignant Pleural Mesothelioma

Systemic chemotherapy with a platinum analogue and pemetrexed has remained the standard first-line treatment for MPM for over two decades, offering only moderate effectiveness. The emergence of immunotherapy, particularly ICIs, has significantly improved treatment outcomes. ICIs function by disrupting the immunosuppressive interactions between checkpoint molecules expressed on tumor cells—such as PD-L1—and their corresponding receptors (e.g., PD-1 or CTLA-4) on immune cells. This interruption reactivates T-cell-mediated immune responses against the tumor, thereby enhancing antitumor immunity [113].

The combination of nivolumab (anti–PD-1) and ipilimumab (anti–CTLA-4) has been approved by the US Food and Drug Administration (FDA) as a first-line treatment for unresectable MPM. This approval was based on the phase III RCT CheckMate 743, which demonstrated a statistically significant improvement in OS for the immunotherapy group compared to the chemotherapy group (pemetrexed plus a platinum analogue, either cisplatin or carboplatin), which constituted 18.1 versus 14.1 months. The trial also revealed important differences in clinical outcomes based on histologic subtype. Among patients with epithelioid tumors, median OS was 18.7 months in the ipilimumab–nivolumab group versus 16.5 months in the chemotherapy group. In contrast, patients with nonepithelioid tumors (sarcomatoid or biphasic) derived greater benefit from immunotherapy, with median survival of 18.1 months versus 8.8 months in the chemotherapy group [114].

Several clinical practice guidelines have incorporated this combination regimen into recommended treatment strategies for MPM, although the recommendations differ somewhat. The National Institute for Health and Care Excellence recommends ipilimumab and nivolumab as one of the treatment options for previously untreated, unresectable MPM in patients with an Eastern Cooperative Oncology Group performance status of 0 or 1 [115]. In contrast, the ASCO guidelines differentiate based on histologic subtype: ipilimumab–nivolumab is the preferred treatment for patients with previously untreated nonepithelioid (sarcomatoid or biphasic) mesothelioma, whereas for those with epithelioid mesothelioma, either chemotherapy (platinum analogue plus pemetrexed) or immunotherapy with ipilimumab–nivolumab is considered an appropriate first-line option [99].

Chemoimmunotherapy regimens have also been explored for patients with MPM. Pembrolizumab, a PD-1 inhibitor, was evaluated in combination with chemotherapy (pemetrexed and a platinum analogue) versus chemotherapy alone in the phase II/III IND.227/KEYNOTE-483 trial. The combination therapy demonstrated a statistically significant improvement in OS, reducing the risk of death by 21% compared to chemotherapy alone. Specifically, the median OS was 17.3 months in the combination therapy group versus 16.1 months in the chemotherapy-alone group. The 3-year OS rate was 25% for the combination therapy group and 17% for the chemotherapy-alone group. Grade 3–4 adverse events occurred in 27% of patients receiving the combination regimen compared to 15% of those receiving chemotherapy alone and were generally manageable [116]. Based on these results, the FDA approved pembrolizumab in combination with pemetrexed and platinum-based chemotherapy in 2024 as a first-line treatment for adults with unresectable, advanced, or metastatic MPM [117].

Another PD-L1 inhibitor that has been evaluated in combination with chemotherapy for mesothelioma is durvalumab. In a phase II RCT, Forde et al. assessed the efficacy and safety of durvalumab in combination with pemetrexed and a platinum agent in previously untreated patients with unresectable MPM. The study met its primary endpoint, demonstrating a median OS of 20.1 months, compared to a historical control of 12.1 months with chemotherapy alone. The safety profile was favorable, with all immunotherapy-related adverse events reported as grade 2 or lower [118]. Similarly, Nowak et al. conducted a phase II trial evaluating the same combination regimen. This study met its primary endpoint as well, with 57% of patients achieving PFS at 6 months [119].

Tremelimumab, a CTLA-4 inhibitor, was evaluated in combination with durvalumab as a neoadjuvant therapy for resectable MPM by Lee et al. Compared with durvalumab alone, patients who received the combination therapy had a longer median OS. However, the primary endpoint of the study was not met, which may be due to the small sample size (20 patients in total), limiting the generalizability of the findings [120]. The combination of tremelimumab and durvalumab was also assessed in a retreatment setting. In this cohort, the median OS from the start of retreatment was 12.5 months, with 1- and 2-year survival rates of 52.9% and 23.5%, respectively. Notably, no grade 3–4 immunotherapy-related adverse events were reported [121].

Messori et al. conducted a meta-analysis of first-line treatments for MPM, comparing novel therapies with standard chemotherapy regimens. The authors extracted data from original RCTs and evaluated clinical effectiveness primarily based on OS. Among the treatment regimens assessed, only the combinations of nivolumab with ipilimumab, bevacizumab with pemetrexed and cisplatin, and chemotherapy with pembrolizumab demonstrated a statistically significant improvement in OS compared with control treatments [117]. Table 4 summarizes targeted therapy and immunotherapy regimens for mesothelioma that have been integrated into current clinical practice.

### 6.2. Targeted Therapy and Immunotherapy of Metastatic Pleural Disease

In contrast to MPM, there is a relative paucity of studies investigating targeted therapies and immunotherapies in patients with metastatic pleural disease. This is primarily because metastatic pleural involvement often represents end-stage cancer, typically presenting as MPE, where the therapeutic focus shifts to palliative interventions such as thoracentesis and pleurodesis, aimed at symptom relief and improving patients’ quality of life [16]. Nevertheless, several biomarkers—such as VEGF, EGF, PD-1—may serve as potential targets for novel therapies, including targeted agents and immunotherapies.

Several anti-VEGF agents have been investigated for the treatment of MPE secondary to metastatic cancers. Bevacizumab has been evaluated in patients with NSCLC via both intravenous and intrapleural routes. A phase II trial assessed systemic bevacizumab combined with chemotherapy (carboplatin and paclitaxel), reporting an overall response rate of 60.8%, a median PFS of 7.1 months, and an OS of 11.7 months [122]. Another phase II RCT evaluated systemic bevacizumab with carboplatin and pemetrexed, showing that 93% of patients achieved MPE control without requiring pleurodesis [123]. Nie et al. compared intravenous and intrapleural administration of bevacizumab, finding a higher response rate with intrapleural delivery (80%) compared to intravenous administration (66.7%). The median duration of response was also longer in the intrapleural group (4.5 vs. 3.7 months) [124]. Several additional RCTs have assessed the effectiveness of intrapleural bevacizumab in combination with systemic chemotherapy, prompting a meta-analysis by Zongwen et al. This meta-analysis demonstrated a significantly improved overall response rate, reduced incidence of chest pain, and better relief of dyspnea in patients receiving the combination therapy compared to chemotherapy alone. Importantly, intrapleural bevacizumab did not significantly increase the incidence of adverse effects, indicating that the combination was well tolerated [125].

Ramucirumab, an anti-VEGF monoclonal antibody, was evaluated in combination with systemic chemotherapy (docetaxel) in a phase II RCT involving patients with MPE secondary to NSCLC who had previously received platinum-based chemotherapy. The primary endpoint—MPE control at 8 weeks—was achieved in 100% of patients [126]. However, the study was limited by its small sample size (*n* = 15) and warrants further investigation. Several antiangiogenic epidermal growth factor receptor tyrosine kinase inhibitors (EGFR-TKIs) have also been assessed in this context. A phase II trial of vandetanib in NSCLC patients with MPE showed that the drug was well tolerated but did not significantly reduce the time to pleurodesis [127]. Cediranib was evaluated in a phase II trial involving patients with malignant ascites or MPE of various primary tumor origins. Cediranib significantly prolonged puncture-free survival and demonstrated a favorable safety profile [128].

A variety of immunotherapeutic agents have been explored in the treatment of MPE since the 1990s, including interleukin-2, TNF-α, and interferons. Although some early-phase studies suggested potential benefits in controlling MPE, subsequent larger trials did not confirm these findings. With the emergence of modern immunotherapy, particularly ICIs, interest in these earlier cytokine-based therapies has diminished [129]. More recent studies have focused on ICIs in patients with MPE secondary to NSCLC. A retrospective multicenter study of 155 previously untreated NSCLC patients with MPE found that ICI combined with chemotherapy significantly improved PFS compared to chemotherapy alone (7.4 vs. 5.7 months, *p* = 0.008), although OS was not significantly different (34.2 vs. 28.3 months, *p* = 0.317) [130]. Another retrospective cohort study comparing ICI-chemotherapy combination therapy to pembrolizumab monotherapy in nonsquamous NSCLC patients with MPE concluded that the combination regimen was more effective [131].

Several trials have also evaluated the intrapleural administration of ICIs. A multicenter phase II study assessed intrapleural nivolumab in patients with metastatic NSCLC and large-volume MPE requiring drainage. The primary endpoint—3-month recurrence-free survival—was not met, with 61.5% of patients experiencing recurrence within 3 months [132]. An exploratory phase I study investigated intrapleural sintilimab in NSCLC patients with moderate to large MPE. The primary endpoint, pleurodesis success rate at 5 weeks, was achieved in 80% of patients, and all participants achieved stable disease. No immune-related adverse events were reported, indicating a favorable safety profile [133].

A number of emerging immunotherapy approaches have been investigated in small-scale studies involving patients with MPE. Oncolytic viruses such as herpes simplex virus 1716 were evaluated in patients with MPM, achieving disease stabilization in 50% of patients following intrapleural administration [134]. Another oncolytic virus, GL-ONC1, was tested in a phase I trial in patients with MPE secondary to MPM, NSCLC, breast cancer, or other solid tumors. GL-ONC1 was administered intrapleurally at varying concentrations without concurrent chemotherapy. The authors concluded that patients with mesothelioma derived the most benefit, possibly due to the disease’s localization within the pleural cavity [135]. Vaccination with autologous dendritic cells was explored in eight patients with MPE secondary to lung cancer, resulting in disease stabilization in 25% of participants [136]. Another promising strategy involved the use of tumor-infiltrating lymphocytes (TILs). In a small-scale study comparing TIL therapy to cisplatin in patients with MPE or malignant ascites, the TIL group demonstrated higher overall response and disease control rates (33.33% and 83.33%, respectively) compared with the cisplatin group (28.57% and 71.43%, respectively). PFS was also significantly prolonged in the TIL-treated group (66.67% vs. 28.57%) [137]. Given the limited sample sizes and exploratory nature of these trials, larger studies are necessary to confirm their efficacy and safety. Table 5 summarizes targeted and immunotherapy regimens for metastatic pleural disease that have shown clinical effectiveness.

## 7. Intrapleural Chemotherapy

Intrapleural infusion therapy for MPE is a localized treatment modality aimed at reducing fluid reaccumulation, inducing pleurodesis, and exerting local anti-tumor effects. Initially limited to the direct instillation of therapeutic agents into the pleural cavity, this approach has evolved into more sophisticated techniques that incorporate physical adjuncts, such as hyperthermia and pressurization. These advancements represent a paradigm shift toward physically enhanced intrapleural therapies, which aim to optimize drug delivery and therapeutic efficacy by modifying the local treatment environment.

### 7.1. Hyperthermic Intrathoracic Chemotherapy

HITOC involves the intraoperative circulation of heated chemotherapeutic agents within the pleural cavity, most commonly cisplatin. The elevated temperature, typically maintained between 40 °C and 43 °C for 60 min or longer, enhances local drug penetration while limiting systemic absorption [138]. Ex vivo experiments have demonstrated that following a 60-min lavage with cisplatin at 42 °C and a concentration of 0.05 mg/mL, the in-tissue drug concentration reaches approximately 2.4 µg/mL at a depth of 1 mm, 1.4 µg/mL at 2 mm, and 0.5 µg/mL at 4 mm. These levels are considered therapeutically adequate, especially given that the bulk of the tumor is typically resected prior to HITOC, which is intended to target residual microscopic disease [139]. The first report of HITOC was published in 1994, and since then, most studies on this modality have been small-scale and non-randomized clinical trials [47,140]. However, several systematic reviews and meta-analyses have been published, offering broader insights into its efficacy and safety [141,142,143,144].

MPM is the most common indication for HITOC and was the first form of MPD for which this modality was implemented. Cisplatin is the most frequently used chemotherapeutic agent, typically tested at intrathoracic doses ranging from 50 to 250 mg/m^2^. A phase I/II study by Richards et al. established 225 mg/m^2^ as the maximum tolerated dose (MTD), as dose-limiting renal toxicity was observed at 250 mg/m^2^. This study reported a median OS of 13 months across the cohort, with significant differences between the low-dose (50–150 mg/m^2^) and high-dose (175–250 mg/m^2^) cisplatin groups. Patients in the high-dose group demonstrated significantly longer median OS (18 vs. 6 months, *p* = 0.0019) and recurrence-free intervals (9 vs. 4 months, *p* < 0.0001) [145]. HITOC using cisplatin in similar dosages has also been applied in selected cases of MPD secondary to other malignancies, including thymic tumors [146], lung cancer [147], and metastatic breast and ovarian cancers [141].

Cisplatin has also been used in combination with other chemotherapeutic agents, most notably doxorubicin. Klotz et al. evaluated this regimen in patients with mesothelioma and reported a median OS of 16.1 months following surgery combined with HITOC [148]. Ried et al. conducted a retrospective study involving a large cohort of 350 patients with MPM or MPD secondary to thymic or other malignancies, with a focus on the safety profile of cisplatin–doxorubicin compared to cisplatin alone. The overall rate of postoperative renal insufficiency was 12%, with 1.4% of patients requiring temporary dialysis. High-dose cisplatin (>125 mg/m^2^) was associated with a 2.7-fold higher risk of renal insufficiency compared to low-dose cisplatin (≤125 mg/m^2^). However, even at high doses, the risk remained within an acceptable range [149]. Markowiak et al. retrospectively studied patients with MPD secondary to thymic carcinoma and reported that the addition of doxorubicin to cisplatin did not improve the 3-year survival rate: 84.6% in the cisplatin-alone group vs. 75% in the cisplatin–doxorubicin group. However, the sample size was small (29 patients), and larger studies are warranted to clarify the efficacy of this combination [150].

Epirubicin is another chemotherapeutic agent that has been evaluated in combination with cisplatin for administration via HITOC. Ambrogi et al. reported a 10-year experience using HITOC with a cisplatin–epirubicin combination following surgical treatment of patients with mesothelioma. The authors observed no cases of systemic toxicity, with a median OS of 22 months and disease-free survival rates of 62% at one year, 37.5% at two years, and 18.5% at five years [151]. Aprile et al. presented findings from another retrospective study that employed HITOC with a cisplatin–epirubicin combination following surgery for MPD secondary to thymic cancer, comparing outcomes to surgery alone. Patients treated with surgery and HITOC experienced higher perioperative morbidity compared to those treated with surgery alone (33.3% vs. 23.1%, *p* = 0.71), but also demonstrated a significantly longer local disease-free interval (88.0 months vs. 57.0 months, *p* = 0.046) [152]. To the best of our knowledge, no study to date has specifically evaluated the added value of epirubicin in cisplatin-based HITOC by directly comparing different chemotherapeutic regimens, although such comparative studies could provide further insights into the efficacy of this combination.

Burt et al. reported the results of a phase I trial aimed at determining the MTD of gemcitabine in combination with cisplatin administered via HITOC following surgery for mesothelioma. All patients received cisplatin at a fixed dose of 175 mg/m^2^, while gemcitabine was administered in escalating doses, starting at 100 mg/m^2^ with 100 mg/m^2^ increments. The MTD was established at 1000 mg/m^2^, as a dose-limiting toxicity (grade 3 leukopenia) occurred at 1100 mg/m^2^ [153]. These findings contrast with those of Milano et al., who conducted a phase II trial using cisplatin at 100 mg/m^2^ and gemcitabine at 1250 mg/m^2^ via HITOC in MPM patients, reporting no major chemotherapy-related adverse events. This discrepancy may be attributed to the small sample size in Milano et al.’s study, which included only five patients [154].

Several other chemotherapeutic agents have been investigated for use in HITOC. Mitomycin C, in combination with cisplatin, was evaluated in a retrospective cohort study involving patients with both MPM and metastatic pleural disease. Grade 3–4 toxicities occurred in 14.3% of patients, while the 9-month overall survival rate was 71.4%, and the recurrence-free survival rate was 57.1% [155]. Paclitaxel has also been studied, either in combination with cisplatin and doxorubicin or as a monotherapy, in a small cohort of patients with MPD secondary to ovarian cancer. Pleural disease progression was observed in one patient (25%) following an 18-month period of disease stability [156].

In patients with simultaneous pleural and peritoneal effusion, diaphragmatic involvement is common. In this context, hyperthermic chemotherapy can be administered concurrently in the intraperitoneal and intrapleural cavities. Djelil et al. reported such a case series involving partial diaphragm resection, with HITOC and hyperthermic intraperitoneal chemotherapy using oxaliplatin, cisplatin or mitomycin. No pleural recurrence was observed over a median follow-up of 88 months, and the authors concluded that the combined approach is feasible and well tolerated [157].

Although HITOC appears to be a promising approach for patients with metastatic pleural disease, the optimal chemotherapy regimen remains unclear. The value of combination regimens is also uncertain, largely due to the predominance of phase I/II trials and retrospective cohort studies, which may be a consequence of the rarity of these conditions. A meta-analysis of HITOC in MPM patients concluded that this approach offers survival benefits in terms of OS [142]. In contrast, a separate meta-analysis focusing on MPD secondary to breast and ovarian cancers found insufficient evidence to determine a survival advantage associated with HITOC. Moreover, symptomatic control of MPE was reported to be inferior compared to other pleurodesis techniques [141]. Larger RCTs are needed to better understand the clinical benefits of HITOC in this patient population. Table 6 summarizes the HITOC regimens used for MPE.

### 7.2. Pressurized Intrathoracic Aerosol Chemotherapy

PITAC is a novel technique first introduced in 2012, adapted from pressurized intraperitoneal aerosol chemotherapy (PIPAC) used for peritoneal metastases. It enables the delivery of chemotherapeutic agents as aerosols under pressure directly into the pleural cavity. Preclinical studies have demonstrated that this method allows for more uniform drug distribution and deeper tumor penetration compared to intrapleural infusions, suggesting its potential as a promising treatment modality for patients with MPE [158]. However, clinical evidence on the efficacy and safety of various chemotherapeutic regimens delivered via PITAC remains extremely limited, with only a few studies conducted to date.

Similar to HITOC, most PITAC studies employed platinum-based agents, with cisplatin being the most frequently used, occasionally substituted with carboplatin [159] or oxaliplatin [160], depending on the primary tumor’s origin. The combination of cisplatin with doxorubicin is among the most commonly used regimens. Drevet et al. described the PITAC technique using cisplatin and doxorubicin in patients with MPE of various origins, provided there were no extrapleural metastases. They recommended repeating PITAC every six weeks, if needed, in combination with systemic chemotherapy [161]. Giger-Pabst et al. reported PITAC outcomes in a small cohort of patients with MPM; the median OS was 26.6 months, and two out of five patients (40%) demonstrated stable disease over a median follow-up of 14.4 months [162].

Kuchen et al. conducted a phase I/II study evaluating the efficacy and safety of PITAC in patients with metastatic pleural disease of various origins. The study reported a reduction in the extent of pleural carcinomatosis score in 57.1% of patients, a decrease in the Ki-67 proliferation index in 33.3%, and a reduction in pleural effusion volume in 38.9% of procedures. The overall rate of adverse events was 14.7%, with no grade 4 toxicities observed [159]. However, the study did not compare outcomes between the cisplatin/doxorubicin and oxaliplatin treatment arms, an important limitation that future studies should address. An additional phase I study is currently underway to assess the safety of PITAC using either cisplatin/doxorubicin or oxaliplatin in patients with MPE. In this trial, oxaliplatin is reserved for patients with MPD secondary to colorectal cancer metastases, while cisplatin/doxorubicin is administered for all other etiologies [160].

Unlike HITOC, there is currently no established MTD for chemotherapeutic agents administered via PITAC. The ongoing phase I clinical trial (NCT06281860) aims to address this gap by determining the MTD of aerosolized cisplatin, which will be administered in escalating doses ranging from 7.5 to 70 mg/m^2^. A notable innovation of this study is the incorporation of moderate hyperthermia (39 ± 1 °C) during PITAC, intended to enhance drug penetration and cytotoxicity in the pleural cavity [163]. To the best of our knowledge, no systematic reviews on PITAC have been published, likely due to the limited number of primary studies available. Table 7 summarizes the chemotherapeutic regimens that have been investigated for PITAC to date.

## 8. Future Directions

To improve outcomes for patients with MPD, future efforts must focus on both diagnostic and therapeutic advancements. In the field of diagnostics, early detection remains a major challenge. At the time of diagnosis, mesothelioma often presents as an advanced-stage disease, and metastatic pleural disease may manifest in end-stage cancer [22]. This underscores the need for improved early diagnostic strategies.

One potential approach is the implementation of targeted screening programs. Currently, no standard or widely recommended screening program exists for MPM, primarily due to the rarity of the disease and the lack of validated early detection tools [31]. However, given that the incidence of MPM continues to rise in certain countries despite global efforts to ban asbestos exposure [164], targeted screening among high-risk populations—particularly individuals with occupational asbestos exposure—may be warranted. Among existing diagnostic tools, low-dose computed tomography (LDCT) has been proposed for use in high-risk cohorts, although its role in MPM screening remains investigational [31]. Another promising area involves the search for reliable biomarkers. Several candidates, including soluble mesothelin-related peptides and fibulin-3, have been evaluated in mesothelioma [165,166]. However, none have demonstrated sufficient sensitivity and specificity for routine clinical screening or early diagnosis. Further research is needed to validate these biomarkers and identify novel molecular indicators of early-stage MPM.

As for MPD of metastatic origin, several established screening programs exist for the primary cancers most commonly associated with pleural metastases. For instance, in lung cancer, LDCT has been validated in large RCTs, demonstrating adequate sensitivity and specificity for detecting early-stage disease [167]. Similarly, routine mammographic screening has significantly improved early detection rates and outcomes for breast cancer [168]. In colorectal cancer, screening using fecal occult blood testing, combined with endoscopic methods such as colonoscopy, has proven effective for early disease detection and reduction in cancer-related mortality [169]. Despite the availability of these effective screening programs, issues related to accessibility and equity remain significant global health challenges. Moreover, even in settings where screening is available and accessible, uptake among the general population often remains suboptimal [170]. Therefore, targeted public health interventions and education campaigns are needed to improve participation rates and ensure that high-risk populations benefit from early detection strategies [171].

In terms of therapeutic advancements, further research is needed to identify biomarkers that can guide treatment selection in patients with MPM. Despite recent progress in chemoimmunotherapy and the use of chemotherapy combined with bevacizumab, clinical outcomes for MPM remain poor due to the tumor’s inherently aggressive nature. Currently, there is insufficient evidence to support the routine use of biomarkers for predicting response to targeted therapies or immunotherapies in MPM [172]. Therefore, ongoing efforts to identify and validate novel predictive biomarkers are essential to optimize the use of existing targeted agents and ICIs. In parallel, the development of new targeted therapies and immunotherapeutic agents remains a key priority to improve survival outcomes in this patient population [39].

While immunotherapy has demonstrated efficacy in NSCLC with pleural involvement, limited data exist for other common causes of MPE such as breast, ovarian, and gastrointestinal cancers. Most current treatment regimens for pleural metastases of non-NSCLC origin are extrapolated from systemic management strategies of the primary tumor, often without dedicated evaluation of pleural response or symptom control in clinical trials. This represents a significant gap in evidence, particularly given the distinct biological behavior and immune microenvironments of pleural metastases compared to their primary sites [173].

Therefore, the development and evaluation of novel targeted and immunotherapeutic agents for MPE secondary to malignancies other than NSCLC remains a critical area for future research. Moreover, some therapeutic agents, such as recombinant human endostatin (Rh-Endostatin, marketed as Endostar), have been investigated primarily in specific geographic regions. For instance, studies assessing the efficacy of Endostar in the treatment of MPE have been conducted exclusively in China, often in combination with systemic chemotherapy or via intrapleural administration. Although a systematic review and meta-analysis of early-phase trials demonstrated promising results in terms of clinical response, disease control, and safety, the lack of broader international validation limits the generalizability of these findings [174].

In addition, it must be acknowledged that most studies evaluating HITOC are small-scale, retrospective in nature, and often lack control groups. Although HITOC has shown potential benefits in managing MPD—particularly in MPM and selected cases of metastatic pleural involvement—the current body of evidence is limited by methodological heterogeneity, small patient cohorts, and short follow-up durations. Only a few phase I/II trials have been conducted, and robust prospective RCTs are lacking [145,146,147,148,149,150,151,152,153,154,155,156]. This limits the ability to draw definitive conclusions regarding the efficacy, safety, and optimal clinical indications for HITOC. The evidence base for PITAC is even more limited, with the majority of existing studies consisting of small, retrospective case series. To better understand the therapeutic value of PITAC—particularly its role in symptom control, survival outcomes, and integration into multimodal treatment strategies—well-designed phase II and III trials are urgently needed. Such studies should also assess the comparative effectiveness of different chemotherapeutic regimens, including combination protocols.

Another possible future direction is the application of HITOC following pleurectomy/decortication via a minimally invasive approach. It has been demonstrated that VATS pleurectomy/decortication is associated with fewer complications and faster recovery compared to open techniques [175,176]. Therefore, combining HITOC with VATS-based pleurectomy/decortication could represent a promising multimodal strategy, potentially offering effective disease control while minimizing surgical morbidity. 

## 9. Conclusions

The management of MPD has significantly advanced over recent decades, driven largely by the refinement of systemic chemotherapy regimens and the emergence of targeted therapies and immunotherapies. Platinum-based chemotherapy, particularly in combination with pemetrexed, remains the cornerstone of treatment for MPM, while the introduction of ICIs—notably the combination of nivolumab and ipilimumab—has further transformed the therapeutic landscape. For metastatic pleural disease originating from lung, breast, ovarian, and other malignancies, systemic chemotherapy tailored to the tumor’s histological and molecular characteristics has yielded meaningful clinical benefits. Nevertheless, due to the aggressive nature and advanced stage at presentation, the therapeutic emphasis often shifts toward palliative measures aimed at symptom control. Emerging therapeutic strategies, including HITOC and PITAC, have shown promise; however, further evidence is needed before these approaches can be integrated into routine clinical practice.

Despite recent progress, several challenges remain unmet. The limited effectiveness and suboptimal toxicity profiles of current treatments underscore the necessity for continued research. Future directions should prioritize biomarker-driven therapies and the development of innovative combination regimens that integrate chemotherapy, targeted agents, and immunotherapy.

## Figures and Tables

**Table 1:** Systemic chemotherapy regimens for malignant pleural mesothelioma.

First Author, Year of Publication [Reference]	Regimen Composition and Dosage	Route of Administration and Therapy Duration
First-line therapies		
Vogelzang et al., 2003 [41]	Pemetrexed 500 mg/m^2^ and cisplatin 75 mg/m^2^	* IV, every 21 days
van Meerbeeck et al., 2005 [42]	Raltitrexed 3 mg/m^2^ and cisplatin 80 mg/m^2^	
Castagneto et al., 2008 [43]	Pemetrexed 500 mg/m^2^ and carboplatin ^ AUC 5 mg/mL/min	
Byrne at al., 1999 [49]	Gemcitabine 1000 mg/m^2^ and cisplatin 100 mg/m^2^	IV, on days 1, 8, and 15, every 28 days
Steele et al., 2000 [52]	Vinorelbine, 30 mg/m^2^	IV, every 7 days
Second-, third-line therapies		
Zauderer et al., 2014 [57]	Vinorelbine, 25 mg/m^2^	IV, on days 1 and 8, every 21 days
	Gemcitabine 1000 mg/m^2^	IV, on days 1 and 8, every 21 days or days 1, 8, and 15, every 28 days
Koda et al., 2021 [58]	Irinotecan 60 mg/m^2^ and gemcitabine 800 mg/m^2^	IV, on days 1 and 8, every 21 days
Metaxas et al., 2020 [59]	Lurbinectedin 3.2 mg/m^2^	IV, every 21 days

^ AUC—area under the curve, * IV—intravenously.

**Table 2:** Systemic chemotherapy regimens for pleural involvement in lung cancer.

First Author, Year of Publication [Reference]	Primary Tumor Histology	Regimen Composition and Dosage	Route of Administration and Therapy Duration
Manegold et al., 2000 [61]	Nonsquamous ^ NSCLC	Pemetrexed 500 mg/m^2^ and cisplatin 75 mg/m^2^	* IV, every 21 days
Scagliotti et al., 2008 [62]		Gemcitabine 1250 mg/m^2^ and cisplatin 75 mg/m^2^	IV, on days 1 and 8, every 21 days
Zukin et al., 2013 [63]		Pemetrexed 500 mg/m^2^ and carboplatin ª AUC 5 mg/mL/min	IV, every 21 days
Scagliotti et al., 2008 [62]	Squamous NSCLC	Gemcitabine 1250 mg/m^2^ and cisplatin 75 mg/m^2^	IV, on days 1 and 8, every 21 days
Pirker et al., 2009 [65]		Vinorelbine 25 mg/m^2^ and cisplatin 80 mg/m^2^	
Sandler et al., 2006 [64]		Paclitaxel 200 mg/m^2^ and carboplatin AUC 6 mg/mL/min	IV, every 21 days
Skarlos et al., 1994 [71]	° SCLC	Cisplatin 50 mg/m^2^ or carboplatin 300 mg/m^2^ and etoposide 300 mg/m^2^	IV, cisplatin on days 1–2, carboplatin on day 1, etoposide on days 1–3, every 21 days
Lara et al., 2009 [76]		Cisplatin 60 mg/m^2^ and irinotecan 60 mg/m^2^	IV, cisplatin on day 1, irinotecan on days 1, 8, and 15, every 28 days
de Jong et al., 2007 [77]		Cyclophosphamide 1000 mg/m^2^ doxorubicin 45 mg/m^2^ and etoposide 100 mg/m^2^	IV, cyclophosphamide and doxorubicin on day 1, etoposide on days 1–3, every 21 days
		Carboplatin AUC 7 mg/mL/min and paclitaxel 175 mg/m^2^	IV, every 21 days
Satouchi et al., 2014 [78]		Cisplatin 50 mg/m^2^ and amrubicin 40 mg/m^2^	IV, amrubicin on days 1–3, cisplatin on day 1, every 21 days

^ NSCLC—non-small cell lung cancer, * IV—intravenously, ª AUC—area under the curve, ° SCLC—small-cell lung cancer.

**Table 3:** Systemic chemotherapy regimens for pleural involvement in non-lung cancers.

First Author, Year of Publication [Reference]	Primary Tumor Site	Regimen Composition and Dosage	Route of Administration and Therapy Duration
Katsumata et al., 2009 [79]	Breast cancer	Doxorubicin 40 mg/m^2^ and cyclophosphamide 500 mg/m^2^,	* IV, every 21 days
		Docetaxel 60 mg/m^2^	
Gennatas et al., 2006 [83]		Gemcitabine 1000 mg/m^2^ and vinorelbine 25 mg/m^2^	IV, on days 1 and 8, every 21 days
González-Martín et al., 2023 [85]	Ovarian cancer	Paclitaxel 175 mg/m^2^ and carboplatin ª AUC 5 mg/mL/min	IV, every 21 days
Kushner et al., 2007 [86]		Docetaxel 35 mg/m2 and carboplatin ª AUC 2 mg/mL/min	IV, on days 1, 8, and 15, every 28 days
Wagner et al., 2012 [87]		* PLD 30 mg m^−2^ and carboplatin AUC 5 mg/mL/min	IV, every 28 days
Kunitoh et al., 2009 [91]	Thymic cancer	Cisplatin 25 mg/m^−2^ and vincristine 1 mg/m^−2^ and doxorubicin 40 mg/m^−2^ and etoposide 80 mg/m^−2^	IV, cisplatin on weeks 1–9; vincristine on weeks 1, 2, 4, 6, and 8; doxorubicin and etoposide on days 1–3 of weeks 1, 3, 5, 7, and 9
Aoki et al., 2014 [93]	^ PMBCL	Cyclophosphamide 750 mg/m^2^, doxorubicin 50 mg/m^2^, vincristine 1.4 mg/m^2^ and prednisolone 40 mg/m^2^	IV, cyclophosphamide, doxorubicin, vincristine, every 21 days Oral prednisolone on days 1–5 of every 21 days

* IV—intravenously, ª AUC—area under the curve, * PLD—pegylated liposomal doxorubicin, ^ PMBCL—primary mediastinal large B-cell lymphoma.

**Table 4:** Targeted therapy and immunotherapy regimens for malignant pleural mesothelioma.

First Author, Year of Publication [Reference]	Pharmacological Group	Regimen Composition and Dosage	Route of Administration and Therapy Duration	Combination Chemotherapy
Targeted therapy				
Zalcman et al., 2016 [94]	* Anti-VEGF	Bevacizumab 15 mg/kg	° IV, every 21 days	Pemetrexed and cisplastin
Pinto et al., 2021 [98]		Ramucirumab 10 mg/kg		Gemcitabine
Immunotherapy				
Baas et al., 2021 [114]	Immune checkpoint inhibitor	Nivolumab 3 mg/kg and ipilimumab 1 mg/kg	IV, Nivolumab, every 14 days; ipilimumab, every 42 days	-
Chu et al., 2023 [116]		Pembrolizumab 200 mg	IV, every 21 days	Pemetrexed and cisplastin

* Anti-VEGF—vascular endothelial growth factor inhibitor, ° IV—intravenously.

**Table 5:** Targeted therapy and immunotherapy regimens for metastatic pleural disease.

First Author, Year of Publication [Reference]	Pharmacological Group	Primary Tumor Histology	Regimen Composition and Dosage	Route of Administration and Therapy Duration	Combination Chemotherapy
Targeted therapy					
Tamiya et al., 2013 [122]	* Anti-VEGF	Nonsquamous ª NSCLC	Bevacizumab 15 mg/kg	° IV, every 21 days	Carboplatin and paclitaxel
Usui et al., 2016 [123]					Carboplatin and pemetrexed
Nie et al., 2020 [124]		NSCLC	Bevacizumab 7.5 mg/kg	^ IP, single time; IV, every 21 days	-
Takemoto et al., 2024 [126]			Ramicrumab 10 mg/kg	IV, every 21 days	Docetaxel
Immunotherapy					
Lv et al., 2021 [133]	Immune checkpoint inhibitor	NSCLC	Sintilimab 100 mg	IP, single time	Platinum analogue and pemetrexed

* Anti-VEGF—vascular endothelial growth factor inhibitor, ª NSCLC—non-small cell lung cancer, ° IV—intravenously, ^ IP—intrapleurally.

**Table 6:** Overview of hyperthermic intrathoracic chemotherapy regimens.

First Author, Year of Publication [Reference]	Primary Tumor Histology	Synchronous Surgery	Regimen Composition and Dosage	Perfusion Time, Temperature
Richards et al., 2006 [145]	* MPM	Pleurectomy/ decortication	Cisplatin 50–250 mg/m^2^	60 min, 42 °C
Klotz et al., 2019 [148]			Cisplatin 200 mg/m^2^ and doxorubicin 100 mg	90 min, 42 °C
Ambrogi et al., 2018 [151]			Cisplatin 80 mg/m^2^ and doxorubicin 25 mg/m^2^	60 min, 42.5 °C
Burt et al., 2018 [153]		Extrapleural pneumonectomy or pleurectomy/ decortication	Cisplatin 175 mg/m^2^ and gemcitabine 100–1100 mg/m^2^	60 min, 40–42 °C
Patel et al., 2019 [155]	MPM, metastatic pleural disease		Cisplatin 50–250 mg/m^2^ and mitomycin C 15 mg	60–90 min, 40–43 °C
Singh et al., 2014 [156]	Metastatic pleural disease due to ovarian cancer	Pleurectomy	Paclitaxel 135 mg/m^2^ and cisplatin 80 mg/m^2^, doxorubicin 15 mg/m^2^ or paclitaxel 175 mg/m^2^ alone	45 min, 42 °C

* MPM—malignant pleural mesothelioma.

**Table 7:** Overview of pressurized intrathoracic aerosol chemotherapy regimens.

First Author, Year of Publication [Reference]	Indication	Synchronous Surgery	Regimen Composition and Dosage	Perfusion Time, Pressure
Drevet et al., 2020 [161]	* MPE of any cause	ª VATS	Cisplatin 10.5 mg/m^2^ and doxorubicin 2.1 mg/m^2^	30 min, 12 mmHg CO_2_
Kuchen et al., 2018 [159]	^ MPD of any cause	Wedge resection	Cisplatin 7.5 mg/m^2^ and doxorubicin 1.5 mg/m^2^ or oxaliplatin 92 mg/m^2^	

* MPE—malignant pleural effusion, ª VATS—video-assisted thoracoscopic surgery, ^ MPD— malignant pleural disease.

## Data Availability

This review article relied on the analysis of data from publicly accessible databases.

## Abbreviations

The following abbreviations are used in this manuscript:

AJCC	American Joint Committee on Cancer
ASCO	American Society of Clinical Oncology
AUC	Area Under the Curve
CHOP	Cyclophosphamide, Doxorubicin, Vincristine, and Prednisolone
CT	Computed Tomography
CTLA-4	Cytotoxic T-Lymphocyte Associated Protein-4
DLBCL	Diffuse Large B-Cell Lymphoma
EGFR	Epidermal Growth Factor Receptor
FAK	Focal Adhesion Kinase
FGF	Fibroblast Growth Factor
FOLFOX	5-Fluorouracil, Leucovorin, and Oxaliplatin
HITOC	Hyperthermic Intrathoracic Chemotherapy
ICIs	Immune Checkpoint Inhibitors
IPC	Indwelling Pleural Catheter
IV	Intravenously
LDCT	Low-Dose Computed Tomography
MPD	Malignant Pleural Disease
MPE	Malignant Pleural Effusion
MPM	Malignant Pleural Mesothelioma
MTD	Maximum Tolerated Dose
NSCLC	Non-Small Cell Lung Cancer
OS	Overall Survival
PDGFR-α	Platelet-Derived Growth Factor Receptor-Alpha
PD-L1	Programmed Death-Ligand 1
PEL	Primary Effusion Lymphoma
PET	Positron Emission Tomography
PFS	Progression-Free Survival
PITAC	Pressurized Intrathoracic Aerosol Chemotherapy
PLD	Pegylated Liposomal Doxorubicin
PMBCL	Primary Mediastinal Large B-Cell Lymphoma
RCT	Randomized Controlled Trial
SCLC	Small Cell Lung Cancer
TILs	Tumor-Infiltrating Lymphocytes
TNF-α	Tumor Necrosis Factor-Alpha
TNM	Tumor, Node, Metastasis
US	United States
VATS	Video-Assisted Thoracoscopic Surgery
VEGF	Vascular Endothelial Growth Factor
WHO	World Health Organization
XELOX	Capecitabine and Oxaliplatin
